# Beyond age: exploring ultimate attainment in heritage speakers and late L2 learners

**DOI:** 10.3389/fpsyg.2024.1419116

**Published:** 2024-08-08

**Authors:** Leonarda Prela, Ewa Dąbrowska, Miquel Llompart

**Affiliations:** ^1^Chair of Language and Cognition, Department of English and American Studies, Friedrich-Alexander-Universität Erlangen-Nürnberg, Erlangen, Germany; ^2^Department of Translation and Language Sciences, Universitat Pompeu Fabra, Barcelona, Spain

**Keywords:** bilingualism, ultimate attainment, heritage language acquisition, late bilinguals, grammar, second language acquisition

## Abstract

According to the Critical Period Hypothesis, successful language learning is optimal during early childhood, whereas language learning outside of this time window is unsuccessful. In this respect, early language acquisition is viewed as convergent and reliable but late acquisition is not. The present study revisits the idea of a critical period by investigating the grammatical attainment of early bilinguals/heritage speakers (HSs), late second/foreign language (L2) learners, and comparable groups of monolinguals by testing Greek-English bilinguals in the two languages they speak by means of a grammaticality judgment task. Our findings show that in English, HSs performed on par with monolinguals, both groups surpassing the late L2 learners, who performed about 2 SDs below the HSs and the monolinguals. In Greek, late L2 learners and monolinguals exhibited comparable performance, contrasting sharply with the HSs’ significantly lower proficiency, which was on average about 5 SDs below the late L2 learners and the monolinguals. Consequently, our results show that the performance gaps between HSs and Greek monolinguals/late L2 learners were more pronounced than the differences between late L2 learners and English monolinguals/HSs, suggesting that the early bilinguals’ success in English may come at the expense of their heritage language (Greek). Furthermore, we observe substantially more individual variation within HSs in their heritage language than within the late L2 learners for their second language. Thus, testing bilinguals in both of their languages allows us to unveil the complexity of grammatical ultimate attainment and prompt a re-thinking of age as the major determining factor of (un)successful attainment.

## 1 Introduction

Understanding the role of age in language acquisition is paramount and remains a focal point of scientific interest up to the present day. Research on ultimate attainment in second language acquisition has revolved around the age factor for decades, with the prevalent view being that early onset is necessary for successful attainment (Johnson and Newport, 1989; Long, 1990; Hyltenstam, 1992). This study embarks on the exploration of the aforementioned point by comparing the grammatical skills from two adult groups of Greek-English bilinguals with different ages of acquisition, and monolingual speakers of these languages. Our two bilingual groups comprise heritage speakers of Greek and late second language learners of English. All participants in our study were strictly matched on age and education and bilinguals were tested in both of their languages.

### 1.1 The role of age and input in language acquisition

One of the most influential ideas in research on language acquisition is the Critical Period Hypothesis (CPH), which postulates the existence of a specific time frame in development during which humans are particularly sensitive to linguistic input (Lenneberg, 1967). According to this view, for language acquisition to be successful, an individual needs to be exposed to a substantial amount of linguistic input during this period. In contrast, individuals who begin learning a second language after the closure of the critical period typically do not attain native-like levels of proficiency (see Johnson and Newport, 1989; DeKeyser, 2000; Abrahamsson and Hyltenstam, 2008, 2009; DeKeyser et al., 2010; Granena and Long, 2013).

However, humans are able to attain reasonable levels of proficiency in a new language even if they are exposed to it in adulthood. To account for this, Bley-Vroman (1989) put forward the Fundamental Difference Hypothesis (FDH), according to which language acquisition early in development is supported by domain-specific learning mechanisms which enable children to learn languages easily and without conscious intention or effort (i.e., implicitly). These domain-specific mechanisms cease to be available after puberty, forcing late learners to resort to domain-general processes when learning a new language. Since these are assumed to be less suited to the task, adult learning requires conscious effort and intention and is generally not fully successful.

In a similar vein, Pullum and Scholz (2002) describe child language acquisition as reliable and convergent as opposed to adult L2 learning, which is unreliable and non-convergent. Child language acquisition is *reliable* in that all typically developing children attain native speaker proficiency if they are exposed to adequate input and it is *convergent* in that all speakers ultimately converge on (more or less) the same grammar. By contrast, adult L2 learners are thought to be incapable of reaching a nativelike end state of grammar and exhibit wide variation in their performance.

The CPH and the FDH thus highlight the role of maturational factors and domain-specific learning mechanisms. In contrast, usage-based (UB) accounts assume that language acquisition in both children and adults involves domain-general processes such as the ability to detect recurrent units in the input, track their frequencies and distribution, infer their meaning from the context and form analogies. Using these processes, learners are able to acquire a network of form-meaning pairings, or constructions, which can be creatively combined to form novel utterances (see Tomasello, 2003; Goldberg, 2009; Bybee, 2010; Ellis et al., 2016).

It follows that the nature of the grammar that is ultimately constructed depends on the one hand, on an individual’s cognitive abilities, and, on the other, on the quantity and quality of the input available to them. Thus, rather than assuming convergence, usage-based models predict the existence of individual differences in learning outcomes. And, in fact, a number of studies have revealed considerable individual differences in monolingual adult native speakers’ knowledge of inflectional morphology (Dąbrowska, 2008; Dąbrowska et al., 2023), the comprehension of various complex syntactic constructions (Dąbrowska, 1997, 2018; Winckel and Dąbrowska, 2024) and the ability to detect various kinds of grammatical anomalies (Llompart and Dąbrowska, 2023; for reviews, see Dąbrowska, 2012and Kidd and Donnelly, 2020).

The existence of individual differences in native speakers’ grammatical attainment raises important methodological issues for second language research. This is because the amount of overlap between the performance of second language learners and native speaker controls depends on the demographic composition of the native sample (Andringa, 2014; Dąbrowska et al., 2020): control groups which include speakers with more varied backgrounds show considerably more variation in performance than groups consisting entirely of highly educated participants, resulting in more second language learners falling within the native range – even when second language learners and controls are matched for socioeconomic status and education. For instance, Dąbrowska et al. (2020) found that 33% of classroom learners and 47% of late immersion learners performed within the native speaker range. These percentages are higher than those reported in most previous studies which used the same or similar stimuli (Johnson and Newport, 1989; DeKeyser, 2000; Birdsong and Molis, 2001), and the authors attribute this to the fact that they used a demographically more diverse control group. A number of other studies which used more varied control groups also report substantial amounts of overlap in performance between late L2 learners and controls (Birdsong, 1992; White and Genesee, 1996; Sasaki, y., 1997; Van Boxtel et al., 2005; Dąbrowska, 2019). In fact, in some cases, high academic achievement L2 learners even outperformed the low academic achievement monolingual native speakers (Dąbrowska and Street, 2006; Street, 2017).

### 1.2 Heritage speakers

Another source of evidence which is potentially problematic for the CPH comes from heritage speakers (HSs), who are broadly defined as individuals who are raised with a home language that is different from the dominant language adopted by the majority of the host community or society (Valdes, 2000). This means that, in most cases, HSs are exposed to their home language from birth (i.e., within the critical period) and grow up speaking that language. If we define one’s native language as the language that “is acquired from naturalistic exposure, in early childhood and in an authentic social context/speech community” (cf. Rothman and Treffers-Daller, 2014, p. 95), HSs are clearly native speakers of their heritage language.

However, HSs differ from monolingual native speakers in terms of both linguistic history and linguistic outcomes. The main reason for this is that, although the two groups often share the same or a similar point of departure in their linguistic journey, their developmental trajectory shifts rather dramatically later on. While monolinguals’ grammatical development continues well into adulthood (Hartshorne et al., 2018), HSs’ native language development is often “arrested” or interrupted (Montrul, 2008). This is most likely due to the fact that HSs receive much less input in the heritage language compared to monolinguals, and that HSs’ language experience is often restricted to the home environment, and hence less varied (Vihman and McLaughlin, 1982; Kohnert et al., 1999). Due to this, several researchers (Andringa, 2014; Cheng et al., 2021; Rothman et al., 2022; Vulchanova et al., 2022) have criticized the composition of control/monolingual groups in heritage language research. They argue that in many studies control monolinguals are recruited from universities and are thus part of a highly educated participant pool. Yet, this same criterion is not necessarily followed in the selection of the HSs, whose heritage language acquisition is “incomplete” (Polinsky, 1997; Montrul, 2002) or, to use more recent terminology, “divergent” (Kupisch and Rothman, 2018). The latter term derives from an effort to destigmatize heritage language acquisition and bilingualism in general. We point to recent works by Kupisch et al. (2017), Kupisch and Rothman (2018), Bayram et al. (2019), and Rothman et al. (2022) for more comprehensive discussions of the matter.

Previous research has shown that heritage speakers often do not attain native-like competence across all areas of language. Instead, their linguistic abilities exhibit traits akin to both monolingual native speakers and late L2 learners, albeit in different respects (Montrul, 2009; Benmamoun et al., 2013). For instance, phonology tends to be an area of relative strength for heritage speakers, as they frequently outperform late bilinguals in various languages (Au et al., 2002; Knightly et al., 2003; Montrul, 2006; Chang et al., 2008; Chrabaszcz and Gor, 2011; Saadah, 2011). Similarly, syntax appears to be a resilient aspect of language (Håkansson, 1995; Montrul, 2006, 2010; Montrul, 2008) although heritage speakers often display inconsistencies between production and comprehension in this domain (Polinsky and Scontras, 2020). On the other hand, morphology (Polinsky and Scontras, 2020) and morphosyntax are characterized as more vulnerable phenomena. In connection to the latter, Au et al. (2002) found no significant advantage for heritage speakers in morphosyntax, with heritage speakers and late bilinguals performing at comparable levels. This suggests that while phonology and some aspects of syntax are robust, morphosyntax may not exhibit the same level of resilience.

The picture gets even more convoluted in studies which involved three-way comparisons (HSs vs. late bilinguals vs. monolinguals). Montrul et al. (2008) conducted a study focusing on Spanish gender agreement. The researchers were interested in the effect of timing and context of acquisition in the ultimate attainment of gender agreement and collected both comprehension and production data. They found that both bilingual groups made systematic gender agreement errors in Spanish.

Thus, despite their early exposure to Spanish, the HSs did not show an advantage over late bilinguals. However, there was a modality effect, with L2 learners making more errors in production and HSs showing relatively poor comprehension. Similarly, Polinsky (2008) looked at gender agreement in HSs of Russian residing in the USA who had English as their dominant language. The results revealed that HSs were significantly outperformed by a group of Russian monolinguals despite their very early exposure to the heritage language. However, due to the small sample size of the study (12 participants), the generalizability of these findings is unclear. More recently, Romano (2020), attempted to (re)examine, among others, the age factor in language acquisition by collecting data from adult speakers (HSs, L2 learners and monolinguals) of Italian. He tested the participants’ mastery of the syntactic and morphological knowledge of Italian clitics. The participants’ knowledge was tested by means of an oral structural priming task and a speeded grammaticality judgment task (henceforth GJT). The age-of-exposure advantage for the HSs in comparison to the L2 learners arose for syntax but not for morphology. The author concluded that HSs resemble first language (L1) speakers in terms of representation of syntactic structures, but they are more similar to L2 learners when it comes to attainment of morphological forms. This result is congruent with previous findings which suggest that inflectional morphology poses difficulties for both late bilinguals and HSs (Montrul, 2016; Uygun et al., 2021).

Note that the mixed findings reviewed above could be due to task and/or modality effects. For instance, some studies report that HSs do better on tasks tapping implicit knowledge while late bilinguals perform better in tasks tapping explicit knowledge (Montrul et al., 2008, 2014; Bowles, 2011). Related to this, late bilinguals tend to perform better in untimed GJTs, most likely because the lack of time pressure enables them to access explicit knowledge about language, while heritage speakers are less affected by time pressure since they lack explicit knowledge of grammar and rely more on linguistic intuitions (Montrul et al., 2008; Bowles, 2011; Montrul, 2016). On the other hand, presenting stimuli in written form tends to disadvantage heritage speakers, who typically have low literacy skills in the HS, while it helps late bilinguals (cf. Dąbrowska et al., 2020).

Thus, in the study described here, we opted for an untimed aural GJT. This follows a number of influential CPH studies (e.g., Johnson and Newport, 1989; DeKeyser, 2000; DeKeyser et al., 2010), and enables both groups to demonstrate their full potential.

### 1.3 Current study

The aim of the current study is to explore the morphosyntactic abilities of two groups of Greek-English bilinguals, namely HSs of Greek (or early bilinguals) and late L2 learners of English (or late bilinguals), in combination with data from monolingual native speakers of Greek and English. These two groups of bilinguals can offer a particularly good testing ground for the effects of early vs. late bilingualism onset. On the one hand, we have the HSs who are exposed to both Greek and English from early on. On the other hand, the L2 learners grew up in a Greek-speaking environment. Their initial exposure to English occurred in instructional settings and was fairly limited; it increased substantially when they moved to the UK. Thus, while the HSs’ exposure to Greek declined over time, the L2 learners’ exposure to English gradually increased (see Supplementary Figures S1, S2).

An additional significant contribution of the current study is that it will provide insights into bilinguals’ language skills by not only combining data from all four groups (i.e., two bilingual and two monolingual groups) but also testing the bilingual participants on the same task in both languages. Such a design affords a unique opportunity to provide a more comprehensive account of bilinguals’ linguistic knowledge, especially considering that most previous research has either compared bilingual against monolingual speakers in the bilinguals’ non-dominant language (Cook, 1997) or has compared bilingual groups against each other in only one of the languages (Lee, 2011; Alarcón, 2020). Crucially, in order to do so, we have applied strict matching criteria for all groups, and we have conducted between-group and between-language comparisons.

The theoretical approaches discussed earlier, namely the CPH and the FDH on the one hand and UB models on the other, both predict native-like performance for late L2 learners in Greek and the early bilinguals in English (since they were exposed to these languages during the critical period *and* used them in most daily settings throughout their lives). Furthermore, all three theories predict that late L2 learners will be non-native like in some respects, although for different reasons (for the CPH and the FDH, this would be due to lack of exposure during the critical period, while UB models would emphasize the quantity and quality of the input and L1 interference). However, the predictions for the outcomes for heritage speakers in Greek are different. According to the CPH and the FDH, HS performance in Greek should be native-like, given that they were exposed to this language during the critical period and continued to use it, albeit less than English, throughout their lives. UB approaches, on the other hand, predict considerable individual differences and nonnative-like levels of proficiency. This is due to the fact that their linguistic experience of Greek is often impoverished in comparison with people who grew up in a Greek-speaking environment, both in terms of quality and quantity: they tend to use Greek primarily in family settings and English at school, at work and most other daily contexts.

## 2 Materials and methods

### 2.1 Participants

We recruited four groups of participants for this study. There were two monolingual groups (Greek and English), who served as controls, and two bilingual groups. The first bilingual group comprised 35 Greek native speakers who were second/foreign language learners of English, with an average age of 39.1 (SD = 7.8). The second group consisted of 31 HSs of Greek, with an average age of 42.4 (SD = 14.2). The HSs were exposed to Greek in naturalistic settings from a very young age [mean age of exposure to Greek was 0.2 years (SD = 0.6)] and continued to use it throughout their lives (Supplementary Figure S2). The late bilinguals arrived in the UK at the age of 28.4 (SD = 7.9) and had been living in the host country for 10.7 years on average (SD = 6.8). Their first exposure to English was at an average age of 8.0 (SD = 2.1) in instructional settings, followed by immersion in the language upon their arrival in the UK. The Greek monolinguals (*n* = 35) resided in Greece, while the English monolinguals (*n* = 35) and both bilingual groups were residents of English-speaking countries. For a comprehensive description of the participants’ characteristics, refer to Supplementary Table S1. Importantly, all groups were matched for age and educational background. None of the participants reported any speech or cognitive disabilities. Recruitment was conducted online via social media and Prolific (an online participant pool) and participants were paid for their participation in the study. Informed consent was granted by all participants. The authors assert that all procedures contributing to this work comply with the ethical standards of the relevant national and institutional committees on human experimentation and with the Helsinki Declaration of 1975, as revised in 2008.

### 2.2 Materials

Participants were first asked to fill in a background questionnaire and complete one (monolinguals) or two (bilinguals) GJTs, with the Greek task preceding the English one. The whole study was conducted online using Gorilla Experiment Builder (Anwyl-Irvine et al., 2020). Participants accessed the experimental platform using either a desktop or a laptop. The duration of the experiment was approximately 40 min for the bilingual participants and about 20 min for the monolinguals.

#### 2.2.1 Background questionnaire

The bilingual participants were asked to supply basic demographic information about themselves and their caregivers as well as about their first exposure to each language and education that they had received in Greek and English. The questionnaire was given in English. The monolingual participants were administered a shorter version of the questionnaire, which included information about age, gender, and education. This data was used for the matching process between the bilingual and monolingual groups. The questionnaire for the bilinguals took around 10 min to complete whereas the one for the monolinguals took around 5 min.

#### 2.2.2 Grammaticality judgment task

Grammatical proficiency in each language was assessed by means of an untimed auditory GJT. The target structures for each language were piloted with native speakers and were selected in such a way that they were potentially challenging even for the monolingual group (for a detailed description see Prela et al., 2022). The task in each language consisted of 120 sentences (half grammatical and half ungrammatical). The English version tested 6 structures (double tense, stranded wh-questions, subcategorization, that-trace, and agreement attraction, control sentences) with 20 sentences for each of them, while the Greek one tested 5 morphosyntactic structures (past perfective tense, grammatical aspect, agreement attraction, adjective-noun, and subject-verb agreement) with 24 sentences for each structure. For examples, please refer to Supplementary Table S2 for English and Supplementary Table S3 for Greek.

The items were presented in a semi-random order with the constraints that items from the same structure could not occur next to one another and that no more than three consecutive (un)grammatical items occurred in a row. The presentation order of the items remained the same across participants. This ensured that, if there were any order effects, these would be the same for all participants. The sentences for the GJTs were recorded by female native speakers of Greek and English respectively, and the audio files were processed to enhance the clarity and quality of the recordings as well as to remove unnecessary pauses.

In each trial in the test, participants were presented with a screen with a written instruction to “Click on Play to listen to the sentence” and they could see a red “Play” button. At the bottom of the screen there were two options, a green tick (for grammatical) and a red cross (for ungrammatical), which the participants had to choose from to indicate the (un)grammaticality of the sentence. At the beginning of the task participants completed two written practice trials (one grammatical and one ungrammatical). Feedback was provided to ensure that participants had understood the task. Afterwards, they were instructed to adjust the volume of their audio system and they were also informed that the rest of the trials would be presented auditorily and that they would only hear each item once. A fixation cross appeared on the screen for 700 ms before every trial. Participants were advised to take a short break in the middle of the task (i.e., after trial 60). Each of the GJTs took approximately 15 min to complete.

## 3 Results

### 3.1 Data pre-processing

Both accuracy and reaction time measures were extracted for the GJTs. The data were pre-processed to ensure that the participants had engaged with the task and had followed the instructions. Firstly, we checked whether participants had listened to the sentences before providing a response. Participants had skipped the audio for 24 trials (i.e., 0.20% of a total of 12,120 trials) in the English GJT and for 12 trials (i.e., 0.10% of a total of 12,120 trials) in the Greek GJT so these items were removed from the analysis. Subsequently, we filtered the reaction time data to identify extreme values. Following the recommendations given by Lachaud and Renaud (2011) and Leys et al. (2013), we used Median Absolute Deviation (MAD) instead of Standard Deviation (SD) to define our threshold. We set a threshold of 3 Median Absolute Deviations (MADs) from the median of the filtered dataset within each group and separately for each language. There were no reaction times (RTs) below the lower threshold (i.e., below 3 MADs) but the total number of trials above the upper threshold was 262 for the Greek GJT (2.16% of all trials) and 622 for the English GJT (5.14% of all trials). These extreme values were removed.

### 3.2 Descriptive statistics

All the datasets used in this article, along with the code and materials required to replicate the reported analyses, can be accessed at: https://osf.io/zgp9x/?view_only=803c8f14217d46c1b3a0756b32cd093f. Table 1 presents a summary of the descriptive statistics (means, standard deviations, ranges, and interquartile ranges) for proportions of correct responses in the Greek and English GJT for each group.

**Table 1:** Proportions of correct responses (mean scores), SDs, ranges and inter-quartile ranges for the GJT for all groups in Greek and English.

	Greek				English			
	Mean	SD	Range	IQR	Mean	SD	Range	IQR
Heritage speakers	0.68	0.14	0.49–0.98	0.58–0.75	0.83	0.08	0.67–0.96	0.76–0.88
Late bilinguals	0.94	0.04	0.86–0.99	0.91–0.96	0.69	0.09	0.49–0.91	0.63–0.74
Greek monolinguals	0.92	0.04	0.83–0.99	0.88–0.95				
English monolinguals					0.83	0.08	0.61–0.94	0.78–0.88

**Figure 1:**
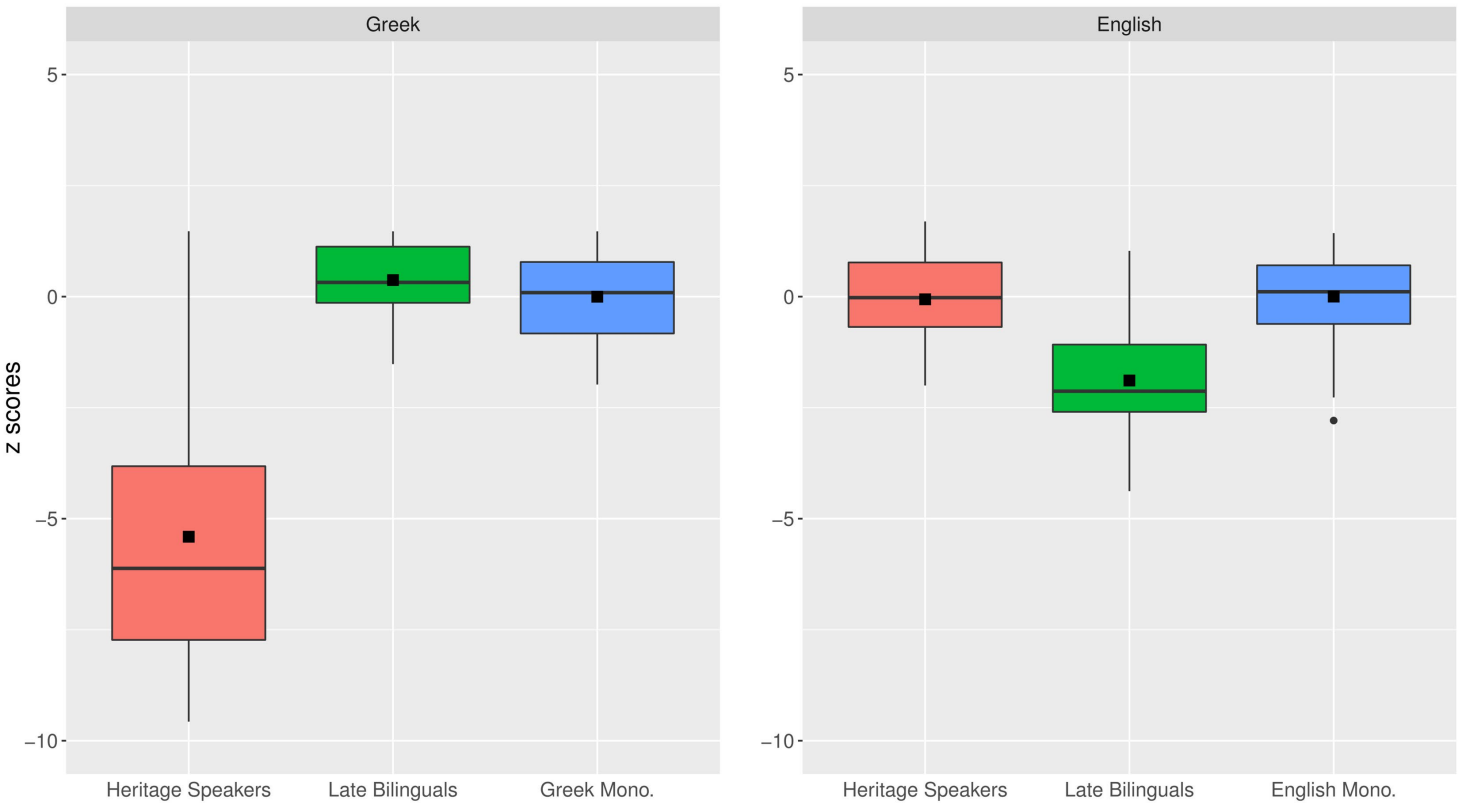
Accuracy on overall performance on **(A)** the Greek (left) and **(B)** the English GJT (right) across the three groups. The y axis shows z-scores computed using the monolingual scale. The black squares indicate mean scores per group.

In order to visualize the within- and between-group differences in overall accuracy, we present the distribution of scores in each group in Figure 1. The numbers on the Y axis are z-scores computed on the monolingual scale; that is to say, the values for all participants were computed by subtracting the mean value for the native monolingual speakers and dividing the difference by the SD for the native monolingual group. As shown in Figure 1, in English, the HSs and English monolinguals are virtually identical, while the late bilinguals’ mean performance is about 2 SDs below the monolingual mean. In Greek, the late bilinguals are very similar to the Greek monolinguals, while the mean value for the HSs is more than 5 SDs below the monolingual mean. It is also worth noting that this group exhibits a vast amount of variation, with the highest-scoring participants performing as well as the highest-scoring monolinguals, whereas the lowest-scoring participant was about 9 SDs below the Greek monolingual mean.

To determine the degree of overlap between the bilinguals (in their two languages) and their monolingual counterparts, we counted the number of participants in each group (bilingual and monolingual) whose performance fell within or above the normal native speaker range (i.e., within 2 SDs of the mean). The native speaker range was computed on the whole native speaker sample for each language separately. The results are presented in Table 2.

**Table 2:** Percentage of participants who performed either within or above and below the normal native speaker range (±2 SDs) by group and language.

	Greek GJT normal range: (100–118)		English GJT normal range: (85–111)	
	Within or above normal range	Below normal range	Within or above normal range	Below normal range
Late bilinguals	35 (100%)	0 (0%)	12 (34.3%)	23 (65.7%)
Heritage speakers	6 (19.4%)	25 (80.6%)	28 (90.3%)	3 (9.7%)
Greek monolinguals	35 (100%)	0 (0%)	NA	NA
English monolinguals	NA	NA	33 (94.2%)	2 (5.8%)

The normal range was 100–118 (out of 120 trials) for the Greek monolinguals and 85–111 (out of 120 trials) for the English monolinguals.

In a population with a normal distribution, about 2.5% of the scores fall 2 SDs below the mean, which is also the case here (0% for the Greek monolinguals and 5.8% for the English monolinguals). Interestingly, although the late bilinguals’ performance in English was lower than that of the English monolinguals, there was a considerable overlap between the two groups, with 34.3% of the late bilinguals falling within the normal native speaker range. However, only 19.4% of the HSs performed within or above the monolingual native speaker range in Greek. This is just over half of the amount of overlap between the late bilinguals and monolinguals in English.

**Figure 2:**
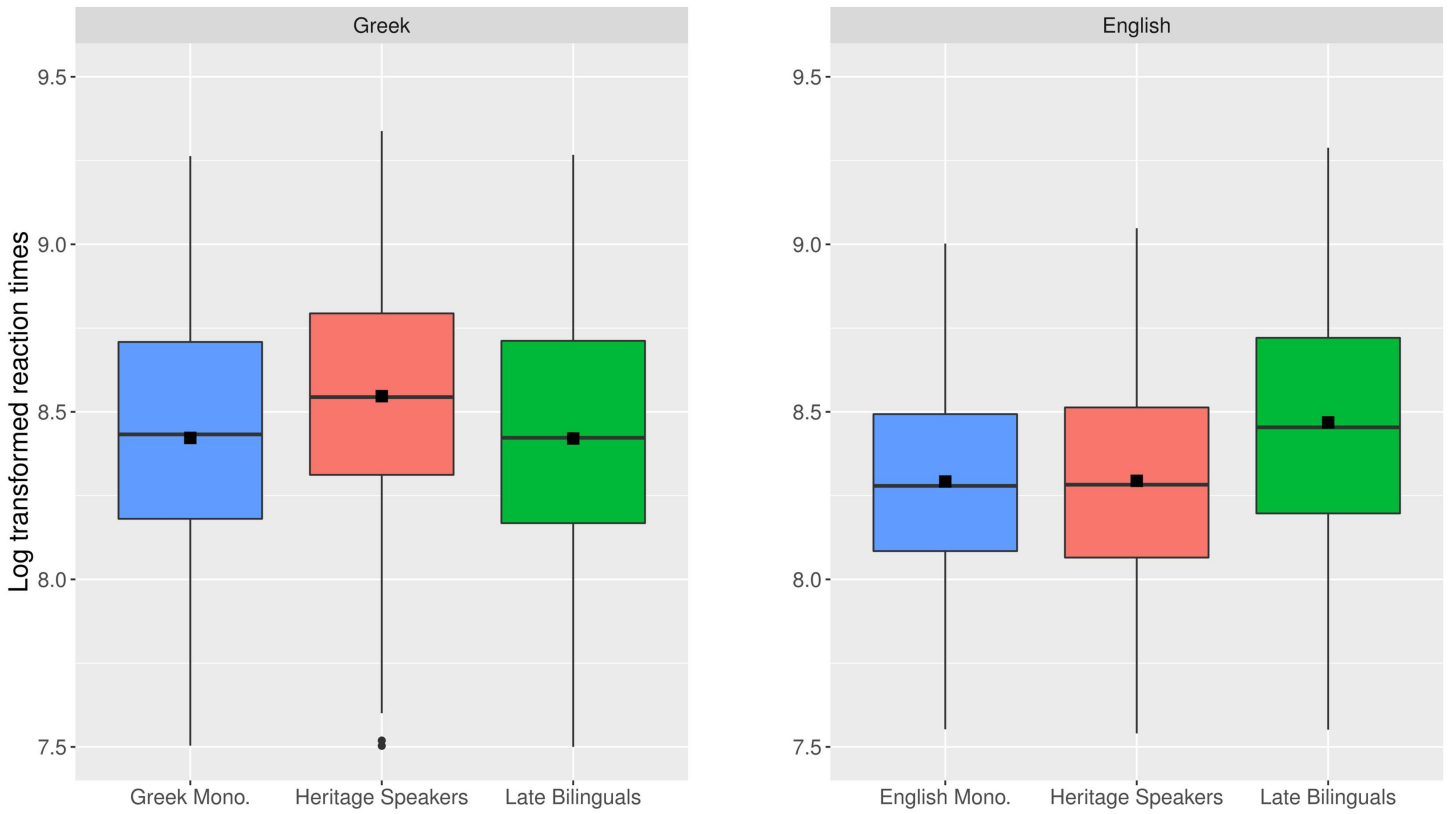
Overall log transformed reaction time performance on **(A)** the Greek (left) and **(B)** the English GJT (right) across the three groups. The black squares indicate mean reaction times per group.

Finally, we present the distribution of reaction times per group in each language (see Figure 2; the descriptive statistics are provided in Supplementary Table S4). As can be observed in the plots, the reaction time data constitute the mirror image of the accuracy results. The HSs are slower than the other two groups in Greek while the late L2 learners are slower than the other two groups in English.

In order to systematically investigate possible differences, we conducted regression analyses for accuracy and RT measures between groups and in the two languages. We initially targeted comparisons that focused on the bilingual groups only and then conducted three-way comparisons, including the monolingual speakers. All analyses were performed in R (R Core Team, 2021).

### 3.3 Statistical analysis: accuracy data

Beginning with accuracy scores, we fitted a generalized linear mixed effects model with a logit linking function (Bates et al., 2015) with response (0 = incorrect, 1 = correct) as the binary dependent variable and *language* and *group*, as well as their interaction, as predictors. The *language* and *group* variables were contrast coded. For *language*, Greek was coded as 0.5 and English as −0.5. For *group*, HSs were coded as −0.5 and late bilinguals as 0.5. As random effects, random intercepts were included for subjects and for items nested within *language*, given that the items were different across languages. By-subject random slopes for the effect of *language* as well as by-item random slopes for the effect of *group* were also included.

The model revealed significant effects of *language, group* and their interaction, thus showing that the two bilingual groups’ performance is different and is additionally modulated by language. In other words, the effect of *language* suggests that overall scores were higher in Greek than in English (this holds true if we compare the accuracy scores of the Greek monolinguals’ performance against that of the English monolinguals, suggesting that the Greek task might have been easier—see Table 1; descriptive statistics). The effect of *group* suggests that late bilinguals achieved higher scores than the HSs. Finally, the interaction indicates that the effect of *group* is modulated by language (see Table 3). This means that the difference in performance between late bilinguals and HSs is not consistent across both languages.

**Table 3:** Model assessing the effects of *language*, *group*, and their interaction on the two bilingual groups’ (HSs and late bilinguals) grammatical performance (accuracy).

Main analysis			
Predictor	*b*	*z*	*p*
*(Intercept)*	2.427	15.583	<0.001
Language	0.971	3.517	<0.001
Group	0.960	4.226	<0.001
Language:Group	4.600	13.326	<0.001

In order to follow up on the significant interaction between *language* and *group*, we split the dataset by language and tested the effect of *group* on grammatical proficiency for each language separately by fitting a logistic mixed effects regression model. The results can be found in Table 4. Here we see effects in the opposite direction. While late bilinguals outperform the HSs in Greek, this difference is reversed for English, where we see that HSs are better than the late bilinguals (see Table 4).

**Table 4:** Follow-up models assessing the effect of *group* in each language (accuracy).

Follow up analysis						
	English			Greek		
Predictor	*b*	*z*	*p*	*b*	*z*	*p*
(Intercept)	0.822	31.88	<0.001	0.680	24.228	<0.001
Group	−0.139	−5.18	<0.001	0.252	8.522	<0.001

Importantly, we were also interested in testing how the two bilingual groups performed in relation to the monolinguals. In order to do this, and since the monolingual groups differ by language, we ran two additional models, one for each language, where we assessed the effect of *group*. Hence, in these analyses, the only fixed effect was *group*, with the monolingual groups mapped onto the intercept, and our random effects included both random intercepts (by-subject and by-item) as well as by-item random slopes for the effect of *group*. By mapping the monolingual groups to the intercept, we were able to draw conclusions about bilingual groups’ performances in relation to the native monolinguals. The English and the Greek model outputs are presented in Table 5.

**Table 5:** Model assessing the effects of *group* (all three groups) in each language (accuracy).

Bilinguals vs. Monolinguals						
	English			Greek		
	*b*	*z*	*p*	*b*	*z*	*p*
(Intercept)	2.575	10.901	<0.001	4.060	14.440	<0.001
HSs	0.160	0.701	0.483	−2.815	−9.081	<0.001
Late bilinguals	−1.348	−5.707	<0.001	0.931	2.933	0.003

For the English model, there was no statistically significant difference between the HSs and the English monolinguals, but the late bilinguals performed significantly worse than the latter. For the Greek model, we observe that the late bilinguals performed slightly better than the Greek monolinguals, whereas the HSs were significantly worse than the monolingual group. As shown here, the difference between HSs and the late bilinguals, judging by the coefficients, is much larger in their native language (Greek). These results are consistent with the patterns shown in Figure 1.

### 3.4 Statistical analysis: reaction time data

Analyses on RT data only included trials for which the participants had responded correctly (81.4% of the trials left after filtering). RT data were log-transformed by means of the default log function in R, which creates a natural logarithm of the value. This was done to reduce the skewness in our data (Cohen et al., 1985; Baayen et al., 2008; Lo and Andrews, 2015). Similar to the procedure followed for the accuracy scores, we conducted two sets of analyses: one with a model that only included the two bilingual groups and then another analysis with by-language models addressing monolingual-bilingual comparisons.

We first fitted a linear mixed effects regression model with *language*, *group* and their interaction as predictors. Again, the *language* and *group* variables were contrast coded as in the accuracy model reported above. The random-effects structure included varying intercepts by subject and by item, the latter nested within *language*, as well as by-subject random slopes for the effect of *language* and random slopes by-item for *group*. The lmerTest package (Kuznetsova et al., 2017) was used to calculate significance and obtain *p*-values for our predictors.

The model revealed significant effects of *language*, and a significant two-way interaction between *group* and *language* but no main effect of *group*. The results (see Table 6) suggest that bilinguals were faster in English than in Greek and the group effect indicates that the late bilinguals did not differ overall from the HSs. However, the interaction suggests that the effect of *group* is modulated by *language*.

**Table 6:** Model assessing the effects of *language*, *group*, and their interaction on the two bilingual groups’ (HSs and late bilinguals) grammatical performance (reaction times).

Main analysis			
Predictor	*b*	*t*	*p*
*(Intercept)*	8.457	414.315	<0.001
Language	0.094	2.522	0.012
Group	0.018	0.740	0.461
Language:Group	−0.317	−8.531	<0.001

Therefore, as for accuracy results, we followed up on this interaction by splitting the dataset by language and testing the effect of *group* on overall grammatical proficiency for each language separately. Results (see Table 7) show that the interaction effect above suggests that the difference in performance observed between late bilinguals and HSs varies across languages. Specifically, the late bilinguals were slower relative to the HSs in English, whereas the HSs were slower than the late bilinguals in Greek.

**Table 7:** Follow-up models assessing the effect of *group* in each language (reaction times).

Follow up analysis						
	English			Greek		
Predictor	*b*	*t*	*p*	*b*	*t*	*p*
(Intercept)	8.323	269.257	<0.001	8.574	280.028	<0.001
Group	0.172	5.442	<0.001	−0.137	−4.601	<0.001

Secondly, we tested the effect of *group* (including all three groups) on overall grammatical performance by conducting separate analyses for each language. The groups in each model were HSs, late bilinguals, and monolinguals in the respective language in order to compare the monolingual with the bilingual groups. The model contained a fixed effect of *group* and the random effects included both by-subject and by-item random intercepts. Finally, we also added by-item random slopes for *group*. The model output is provided in Table 8.

**Table 8:** Model assessing the effects of *group* (all three groups) in each language (reaction times).

Bilinguals and monolinguals						
	English			Greek		
	*b*	*t*	*p*	*b*	*t*	*p*
(Intercept)	8.320	295.736	<0.001	8.430	235.684	<0.001
HSs	0.002	0.094	0.925	0.142	4.524	<0.001
Late bilinguals	0.175	6.256	<0.001	0.004	0.153	0.879

As with the accuracy data, English monolinguals’ and late bilinguals’ performance is statistically different in English, with the late bilinguals exhibiting longer RTs during the English GJT. The HSs perform similarly to the monolinguals. Also, in line with previous findings, for the Greek GJT, we see that the late bilinguals behave like the Greek monolinguals in terms of RTs, but the HSs need significantly more time than the monolinguals to process the GJT sentences in Greek.

Overall, our analyses reveal similar findings across accuracy and RT measures. We observe that both bilingual groups are statistically different from the respective monolinguals in their weaker languages (i.e., Greek for HSs and English for the late bilinguals). In other words, HSs differed from monolinguals in Greek and late bilinguals differed from monolinguals in English. Finally, in terms of accuracy, this difference between the HSs and the Greek monolinguals is much larger than that between the late bilinguals and the English monolinguals.

## 4 Discussion

In this study we set out to explore the performance of two groups of Greek-English bilinguals, heritage speakers and late L2 learners, on tasks assessing morphosyntactic abilities in both languages and compare them to each other and to monolingual controls. This design allows us to offer a more complete account of (bilingual) speakers’ morphosyntactic abilities than most previous studies, which focused on comparing one bilingual group (either HSs or late bilinguals) to baseline data or tested two bilingual groups but only in one of the two languages (cf. De Houwer, 2023). As explained in the introduction, the FDH and the CPH predict that HSs’ performance in Greek should be similar to that of monolingual native speakers since they were exposed to Greek during the critical period (as well as later on in life), while UB approaches predicted substantial departures from the monolingual norm due to impoverished input.

Our data indicate that not all HSs achieve high proficiency despite early exposure to their heritage language. Additionally, HSs exhibit substantial individual variation, indicative of a lack of grammatical convergence. Interestingly, our findings reveal that late bilinguals are more nativelike in English than HSs are in Greek, with late bilinguals demonstrating less variability than expected. In fact, the variability observed in late bilinguals is lower than that in HSs. Despite their delayed onset of language acquisition, late bilinguals exhibit less variation and demonstrate more nativelike performance.

These results challenge the assumption that early exposure, or exposure within a critical period, necessarily leads to reliability and convergence. The ideas of reliability and convergence (Pullum and Scholz, 2002) are rooted in the belief that exposure during early developmental stages guarantees nativelike proficiency. As previously stated, according to the CPH (Lenneberg, 1967) and the FDH (Bley-Vroman, 1989), children grow up to become successful language learners (reliability) whose systems resemble the acquired systems of others in their speech community (convergence). The terms reliability and convergence can also be said to correspond to Bley-Vroman’s (1990) characterization of the difference between early and late learners in terms of success-failure and uniformity-variability.

In the subsequent discussion, we elaborate further on our results for each bilingual group separately, aiming to discuss them within a theoretical framework that offers a more nuanced understanding of these outcomes.

### 4.1 L2 learners

Since the late bilinguals and the Greek monolinguals grew up in a Greek-speaking environment, it is not surprising that they exhibit high levels of performance in this language. Remarkably, the late bilinguals were slightly better than the monolingual group. Although this difference is small, it could mean that the late bilinguals benefit from the metalinguistic awareness that develops through learning a second language in instructional settings or simply that bilingualism has a beneficial effect on language skills overall, as argued by Peal and Lambert (1962), Clark (1978), and Tunmer and Myhill (1984). Additionally, the performance of the late bilinguals was poorer in English than in Greek. To be precise, the late bilinguals’ accuracy scores were around 2 SDs below the monolingual mean in English (see Figure 1). This is explained by the fact that the late bilinguals were first exposed to the language through schooling and have grown up under conditions of reduced input, at least during the first decades of their life before moving to the UK and being fully immersed in English. This comparatively reduced input due to their later bilingualism onset is expected to result in lower performance (Flege and Liu, 2001; Flege, 2009, 2019).

### 4.2 Heritage speakers

Additionally, our results revealed that the performance of the HSs and the monolinguals in English was almost identical. The HSs were exposed to English relatively early (M = 2.2 years), and English was the dominant language outside the home setting. However, the HSs’ performance in Greek was much worse than that of the Greek monolinguals (around 5 SDs below the monolingual mean—see Figure 1). The HSs, like the Greek monolinguals, were exposed to Greek from birth, or very soon after (M = 0.2 years). If early bilingualism onset results in complete mastery (Johnson and Newport, 1989; Long, 1990; Hyltenstam, 1992), early exposure to Greek for the HSs should have resulted in nativelike ultimate attainment in all participants. What we observe instead is that, despite the early exposure and the fact that the overwhelming majority continued to use Greek throughout their entire life (see Supplementary Figure S2), the HSs exhibit vast individual differences in performance. In fact, the HSs’ highest performing participant is similar to the highest scoring monolingual while the lowest performing HS scored as low as around 9 SDs below the monolingual mean. This finding comes in sharp contrast with Pullum and Scholz (2002) who characterize child language acquisition as reliable and convergent. Although the HSs were exposed to Greek in their very early childhood, we observe a very wide variation in their performance.

### 4.3 Comparing HSs and late bilinguals

Another dimension that we wanted to explore in this study was the difference between the degree of divergence between the HSs and the Greek monolinguals on one hand and the late bilinguals and the English monolinguals on the other. Our results show that the within-group individual variation is much wider for the HSs in Greek than for the late bilinguals in English. Additionally, the observed difference between the late bilinguals and the monolinguals in English is much smaller than the difference between the HSs and the monolinguals in Greek (see Figure 1).

These findings can be explained by appealing to the differences in their language histories. As pointed out earlier, the late bilinguals’ exposure to English followed a trajectory with gradually increasing input. Their first contact with English was in instructional settings in Greece and later transitioned to a naturalistic setting through immigration. This might have resulted in performance more similar to the native speakers (HSs and English monolinguals) and less individual variation. The HS results provide us with the other side of the coin. Initially they were fully immersed in Greek but because they were growing up in an English-speaking environment, their input in Greek decreased gradually across the lifespan (Supplementary Figure S2) and occurred primarily in family settings.

### 4.4 Moving beyond age

If age-related factors fail to offer a satisfactory account, it is important to explore alternative explanations, with (quality and quantity of) input emerging as a strong candidate for further investigation. We know that exposure to the heritage language typically decreases over the life span, and we observe a shift in dominance with the majority language taking the lead. Furthermore, HS typically use the heritage language primarily in family settings, while experiencing the majority language in a variety of different contexts (school, work, peers, institutional settings, etc.). As a result, HSs often stop developing the heritage language before achieving native-like proficiency, or even regress in their development (Montrul, 2006, 2008; Polinsky, 2008; Kupisch and Rothman, 2018). This would explain the relatively poor performance of our HS in Greek as a group. Furthermore, although – as emphasized throughout this paper, they continued to use Greek on a regular basis throughout their lives (Supplementary Figure S2), there were considerable differences in both current language use and earlier exposure, and these may be responsible for the observed differences in linguistic outcomes. Further research is necessary to evaluate this proposal.

With regard to the L2 learners, although they were on average less accurate than the English monolinguals, there was remarkable overlap between the two groups’ performance. Specifically, 34.3% of the L2 learners achieved scores within the normal range for native speakers (cf. Table 2). This result is consistent with several earlier studies which report overlap between late bilinguals and native monolinguals (Birdsong, 1992; Ioup et al., 1994; White and Genesee, 1996; Bialystok, 1997; Dąbrowska et al., 2020). Additionally, this finding is remarkable considering that previous research has shown that native speaker grammatical development continues all the way through adulthood (Hartshorne et al., 2018) but our late bilinguals have been living in an English-speaking environment for less than a decade on average. Another noteworthy point is that we witness this amount of overlap in the spoken modality when previous research (Dąbrowska et al., 2020) has demonstrated that this disadvantages late bilinguals.

In general, for the late bilinguals, the results mean that increased exposure (among other factors) can lead to high L2 proficiency and that despite starting later, late bilinguals are still capable of reaching nativelike attainment (at least in the morphosyntactic domain). For the HS, the results mean that they are a subset of native speakers with early bilingualism onset but with a divergent acquisition that is extremely variable, and they may need to be supported either through schooling (e.g., heritage language schools) or by consistent input through interactions and engagement in heritage communities. Overall, our findings suggest a potential alignment with UB approaches, which view language as a dynamic system (Bybee, 2010; Diessel, 2017) that is malleable to external circumstances such as experience. In this school of thought, individual variation within bilingual speakers is normal and is viewed as an indication of the complexity of the bilingual experience rather than a problem (Putnam et al., 2018; Adamou, 2021; Bialystok, 2021; López et al., 2023). By adopting this approach, we emphasize the continuous support that HSs, especially children, need during development but also encourage L2 learning even at later stages of life.

Finally, it is important to underline that the goal of our study is not to compare the groups to each other in order to establish superiority or inferiority. Instead, our objective is to elucidate that (non)nativelike ultimate attainment may not be primarily attributable to a monocausal explanation. By combining data from two groups of bilinguals in both of their languages we have seen that traditional comparisons favor one-sided perspectives. The analysis that we conducted in this study allowed us to add an additional layer into this exploration by looking at both sides of the coin. The investigation into additional factors affecting ultimate attainment constitutes one of our primary interests and the next step of our research endeavors.

## Data availability statement

The datasets presented in this study can be found in online repositories. The names of the repository/repositories and accession number(s) can be found at: https://osf.io/zgp9x/?view_only=803c8f14217d46c1b3a0756b32cd093f.

## Ethics statement

The studies involving humans were approved by the Research Ethics Committees of the Friedrich Alexander University of Erlangen-Nuremberg and the University of Birmingham [ERN_16-0608AP23]. The studies were conducted in accordance with the local legislation and institutional requirements. The participants provided their written informed consent to participate in this study.

## Author contributions

LP: Conceptualization, Data curation, Formal analysis, Investigation, Methodology, Project administration, Visualization, Writing – original draft, Writing – review & editing. ED: Conceptualization, Formal analysis, Funding acquisition, Methodology, Supervision, Writing – review & editing. ML: Conceptualization, Methodology, Supervision, Writing – review & editing.
