# Supplementary material for: Beyond age: exploring ultimate attainment in heritage speakers and late L2 learners
Source: Front Psychol. 2024 Aug 8;15:1419116. doi: 10.3389/fpsyg.2024.1419116 (PMC11340529; doi:10.3389/fpsyg.2024.1419116)
Supplement: Supplementary file 1 [file Data_Sheet_1.PDF]

Table S1: Demographic information on the matched sample.

**Language Groups**		**Age**	**Education**	**AoE[1]_Greek**	**AoE_English**
**Bilinguals**					
L2 learners	*mean*	39.1	17.9	0.1	8.0
	standard deviation	7.8	3.5	0.3	2.1
	min-max	23-52	11-28	0-1	3-12
Heritage speakers	*mean*	42.4	17	0.2	2.2
	standard deviation	14.2	3.3	0.6	2.7
	min-max	19-64	11-24	0-3	0-10
**Monolinguals**					
English monolinguals	*mean*	39.7	16.4		From birth
	standard deviation	10.6	2.7		From birth
	min-max	20-61	11-12		From birth
Greek monolinguals	*mean*	45.0	16.0	From birth	
	standard deviation	13.7	3.0	From birth	
	min-max	19-72	12-23	From birth	

[1] AoE refers to age of first exposure to the language.

Table S2: The types of morphosyntactic structures (on the left) that were tested in the English GJT with corresponding grammatical and ungrammatical examples (on the right). The asterisk preceding a sentence indicates that it is ungrammatical.

**Target Structure**	**Condition**	**Example**
**Control sentences**	Grammatical	Many kids have problems with adjusting to high school.
	Ungrammatical	*Many kids *has* problems with adjusting to high school.
**Double tense**	Grammatical	When did the last election take place in North America?
	Ungrammatical	*When did the last election *took* place in North America?
**Stranded Wh-Questions**	Grammatical	What does your mum believe your aunt meant by that?
	Ungrammatical	*What does your mum believe *what* your aunt meant by that?
**Subcategorization**	Grammatical	My friends and I really enjoy playing football.
	Ungrammatical	*My friends and I really enjoy *to play* football.
**That Trace**	Grammatical	What do you think will be this year's revenue?
	Ungrammatical	*What do you think *that* will be this year's revenue?
**Agreement Attraction**	Grammatical	The structure of the new buildings is fascinating.
	Ungrammatical	*The structure of the new buildings *are* fascinating.

Table S3: The types of morphosyntactic structures (on the left) that were tested in the Greek GJT with corresponding grammatical and ungrammatical examples including glossing. The translations are provided for the ungrammatical items. The asterisk preceding a sentence indicates that it is ungrammatical.

**Target Structure**	**Condition**	**Example**
	Grammatical	To pinasmeno aγori kani parapona sti mitera tu.<br>The hungry boy$^{\text{-3SG.SBJ}}$ complain$^{\text{-PRS.ACT.3SG}}$ to his mother.
**Subject-Verb Agreement**	Ungrammatical	*To pinasmeno aγori kanun parapona sti mitera tu.<br>The hungry boy$^{\text{-3SG.SBJ}}$ complain$^{\text{-PRS.ACT.3PL}}$ to his mother.
	Translation	The hungry boy *are* complaining to his mother.
	Grammatical	To traγiko telos tis tenias prokalese θlipsi stus θeates.<br>The$^{\text{-N}}$ tragic$^{\text{-N}}$ end$^{\text{-N}}$ of movie cause sadness to spectators.
**Adjective-Noun Agreement**	Ungrammatical	*O traγikos telos tis tenias prokalese θlipsi stus θeates.<br>The$^{\text{-M}}$ tragic$^{\text{-M}}$ end$^{\text{-N}}$ of movie cause sadness to spectators.
	Translation	The tragic end of the movie caused sadness to the spectators.
	Grammatical	Tin proiγumeni paraskevi i Katerina estile ena ðema stin fili tis sti θessaloniki.<br>Last Friday Catherine send$^{\text{-PAST.PERF.ACT.3SG}}$ a package to friend in Thessaloniki.
**Past Perfective Tense**	Ungrammatical	*Tin proiγumeni paraskevi i Katerina estalse ena ðema stin fili tis sti θessaloniki.<br>Last Friday Catherine send$^{\text{-PAST.PERF.ACT.3SG}}_{\text{(ungrammatical regularization)}}$ a package to friend in Thessaloniki.
	Translation	Last Friday Catherine *sended* a package to her friend in Thessaloniki.
	Grammatical	Eno o Nikos ke i Katerina χorevan, ksafnika i musiki stamatise.<br>While Nick and Catherine dance$^{\text{-PST.IPFV.ACT.3PL}}$ suddenly the music stop$^{\text{-PST.PRF.ACT.3PL.}}$
**Grammatical Aspect**	Ungrammatical	*Eno o Nikos ke i Katerina χorepsan, ksafnika i musiki stamatise.<br>While Nick and Catherine dance$^{\text{-PST.PRF.ACT.3PL}}$ suddenly the music stop$^{\text{-PST.PRF.ACT.3PL.}}$
	Translation	While Nick and Catherine *danced*, suddenly the music stopped.
	Grammatical	I elipsi trofimon se poles polis tis Venezuelas eχi ftasi se anisiχitika epipeða.<br>The lack$^{\text{-NOM.3SG.SBJ}}$ food$^{\text{-GEN.3SG}}$ in many cities of Venezuela reach$^{\text{-PRS.PRF.ACT.3SG}}$ to alarming levels.
**Agreement Attraction**	Ungrammatical	*I elipsi trofimon se poles polis tis Venezuelas eχun ftasi se anisiχitika epipeða.<br>The lack$^{\text{-NOM.3SG.SBJ}}$ food$^{\text{-GEN.3PL}}$ in many cities of Venezuela reach$^{\text{-PRS.PRF.ACT.3PL}}$ to alarming levels.
	Translation	The lack of food in many cities of Venezuela *have* reached an alarming level.

Table S4: Descriptive statistics of filtered reaction time data for correct trials only including mean scores, SDs, ranges and IQRs for the GJT for all groups in Greek and English.

	**Greek**				**English**			
	Mean	SD	Range	IQ Range	Mean	SD	Range	IQ Range
Heritage speakers	5440.893	1831.888	1022.3-11363.6	4064.300-6591.750	4192.289	1329.541	1487.8-8502.9	3180.575-4975.950
Late bilinguals	4823.713	1836.363	1398.365-10583.03	3487.200-6052.247	5070.858	1871.553	507.3-10809.71	3623.189-6127.550
Greek monolinguals	4806.797	1802.042	1327.8 - 10545.2	3501.05-6026.55				
English monolinguals					4155.943	1224.964	171.2-8120.5	3241.075-4881.000

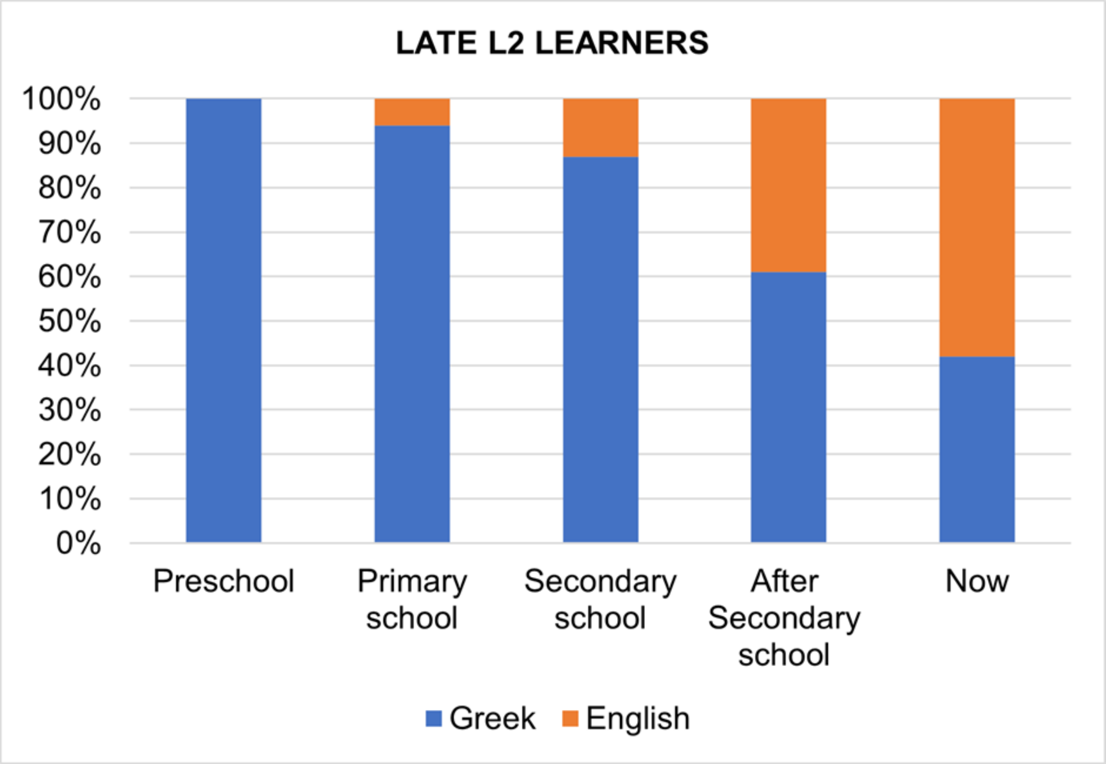

Figure S1: Language use across the life span for the late L2 learners. The percentages indicate the amount of usage of Greek (blue) against English (orange) across five different life periods spanning from birth until the time of testing.

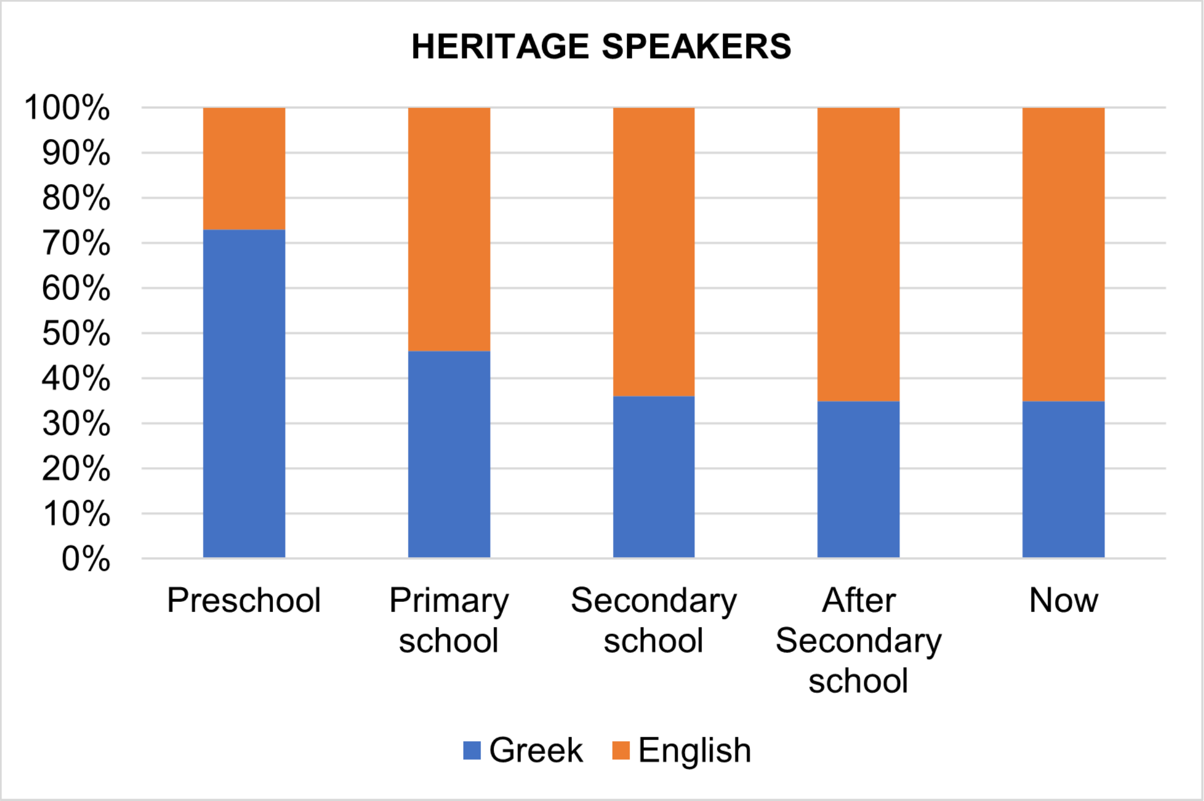

Figure S2: Language use across the life span for the heritage speakers. The percentages indicate the amount of usage of Greek (blue) against English (orange) across five different life periods spanning from birth until the time of testing.
